# Identification of suitable endogenous control genes for microRNA expression profiling of childhood medulloblastoma and human neural stem cells

**DOI:** 10.1186/1756-0500-5-507

**Published:** 2012-09-14

**Authors:** Laura A Genovesi, Denise Anderson, Kim W Carter, Keith M Giles, Peter B Dallas

**Affiliations:** 1 Brain Tumour Research Program, Telethon Institute for Child Health Research, Centre for Child Health Research, University of Western Australia, Perth, Western, Australia; 2 Division of Bioinformatics and Biostatistics, Telethon Institute for Child Health Research, Centre for Child Health Research, University of Western Australia, Perth, Western, Australia; 3 Laboratory for Cancer Medicine, Western Australian Institute for Medical Research, Centre for Medical Research, University of Western Australia, Perth, Western Australia

**Keywords:** MicroRNA, Medulloblastoma, Neural stem cells, Gene expression profiling, Quantitative RT-PCR

## Abstract

**Background:**

Medulloblastoma (MB) is the most common type of malignant childhood brain tumour. Although deregulated microRNA (miRNA) expression has been linked to MB pathogenesis, the selection of appropriate candidate endogenous control (EC) reference genes for MB miRNA expression profiling studies has not been systematically addressed. In this study we utilised reverse transcriptase quantitative PCR (RT-qPCR) to identify the most appropriate EC reference genes for the accurate normalisation of miRNA expression data in primary human MB specimens and neural stem cells.

**Results:**

Expression profiling of 662 miRNAs and six small nuclear/ nucleolar RNAs in primary human MB specimens, two CD133+ neural stem cell (NSC) populations and two CD133- neural progenitor cell (NPC) populations was performed using TaqMan low-density array (TLDA) cards. Minimal intra-card variability for candidate EC reference gene replicates was observed, however significant inter-card variability was identified between replicates present on both TLDA cards A and B. A panel of 18 potentially suitable EC reference genes was identified for the normalisation of miRNA expression on TLDA cards. These candidates were not significantly differentially expressed between CD133+ NSCs/ CD133- NPCs and primary MB specimens. Of the six sn/snoRNA EC reference genes recommended by the manufacturer, only RNU44 was uniformly expressed between primary MB specimens and CD133+ NSC/CD133- NPC populations (*P* = 0.709; FC = 1.02). The suitability of candidate EC reference genes was assessed using geNorm and NormFinder software, with hsa-miR-301a and hsa-miR-339-5p found to be the most uniformly expressed EC reference genes on TLDA card A and hsa-miR-425* and RNU24 for TLDA card B.

**Conclusions:**

A panel of 18 potential EC reference genes that were not significantly differentially expressed between CD133+ NSCs/ CD133- NPCs and primary human MB specimens was identified. The top ranked EC reference genes described here should be validated in a larger cohort of specimens to verify their utility as controls for the normalisation of RT-qPCR data generated in MB miRNA expression studies. Importantly, inter-card variability observed between replicates of certain candidate EC reference genes has major implications for the accurate normalisation of miRNA expression data obtained using the miRNA TLDA platform.

## Background

Medulloblastoma (MB) is the most common malignant paediatric brain tumour, and a major cause of childhood cancer related morbidity and mortality [1]. Several molecular subtypes of MB have been identified on the basis of specific gene expression signatures [2-6], suggesting that different sub-groups may arise from distinct cells of origin. In human MB, a subpopulation of CD133-expressing cells was identified that displayed similar properties to NSCs, including self-renewal and multipotency [7,8]. Additional studies demonstrated that these putative brain tumour stem cells (BTSCs) were capable of initiating tumour formation in immunodeficient mice [9]. These human data combined with evidence from two different murine MB models [10,11] strongly implicate CD133+ NSCs as a cell of origin for a subset of MB.

MicroRNAs (miRNAs) are a class of short, non-coding RNAs that down-regulate gene expression at a post-transcriptional level [12,13]. Functioning as guide molecules for silencing complexes, miRNAs utilise anti-sense complementarity to inhibit the expression of specific messenger RNA (mRNA) targets by either repressing translation and/or inducing deadenylation and subsequent mRNA degradation [13-16]. miRNAs play key regulatory roles in various cellular processes including cell proliferation [17], apoptosis [18] and differentiation [19]. Numerous studies have demonstrated significantly altered miRNA expression patterns in a wide range of human cancer types compared to normal tissues, suggesting that specific miRNAs might act as tumour suppressor genes or oncogenes (Reviewed in [20] and [21]). In addition to their potential as novel molecules for cancer therapy [22], miRNAs represent an emerging class of diagnostic and prognostic markers [23-26].

Adaptations of existing technologies for gene expression profiling including RT-qPCR, chip-based microarrays, and next generation sequencing have enabled the high-throughput profiling of miRNA expression [23,27-31]. The accuracy of these methods is dependent upon correcting for non-biological sample-to-sample variation that could be introduced during the steps from sample preparation to amplification [23]. For miRNA RT-qPCR expression data, several methods have been described to correct for this variation, the most frequent of which is the normalisation to endogenous control (EC) reference genes [32]. An ideal reference gene should be highly expressed, exhibit minimal expression level variation in cells or tissues under investigation, and be of similar length to that of the target gene [33]. Additionally, extraction and quantification efficiency and storage stability of an ideal reference gene should be equivalent to the gene under investigation [33]. However, previous studies have demonstrated that a single universal EC reference gene with these properties for all cell or tissue types is unlikely to exist [34-37]. Normalisation of RT-qPCR data to unreliable reference genes may lead to incorrect quantification of miRNAs of interest [33,38], and the importance of validating suitable candidate EC reference genes in a cell and/or tissue-specific context has been demonstrated previously [33]. Although several research groups have performed miRNA expression profiling studies in primary MB specimens, the validation strategies for the selection of EC genes for normalisation of miRNA gene expression data were not reported [39-45]. Additionally, none of these studies profiled CD133+ NSCs and/or CD133- NPCs, instead normalising miRNA expression to EC gene expression levels in human adult and fetal cerebellum. This study describes a robust strategy for the identification of suitable EC genes for the normalisation of miRNA RT-qPCR data in MB profiling studies. Using the main selection criteria of good measurability and uniform expression, candidate EC reference genes for miRNA data normalisation were investigated in nine primary MB specimens, and two populations of CD133+ NSCs and CD133- NPCs. The uniformity of expression of these candidate EC genes was subsequently investigated across all samples, with the relative quantities of miRNAs, hsa-miR-144*, hsa-miR-21* and hsa-miR-923 assessed using five different normalisers, to determine the impact of EC reference gene selection on relative expression of individual miRNAs of interest.

## Results

### Inter-card variation for candidate small nuclear (sn) and small nucleolar (snoRNA) EC reference genes on the TLDA platform

**Figure 1:**
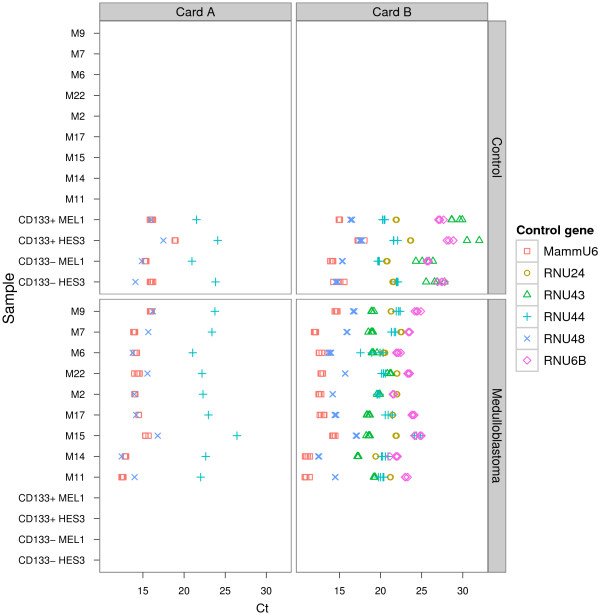
Quantification cycle (Cq) values for the technical replicates of proposed EC reference genes of the (TLDA) cards A (left) and B (right).

**Figure 2:**
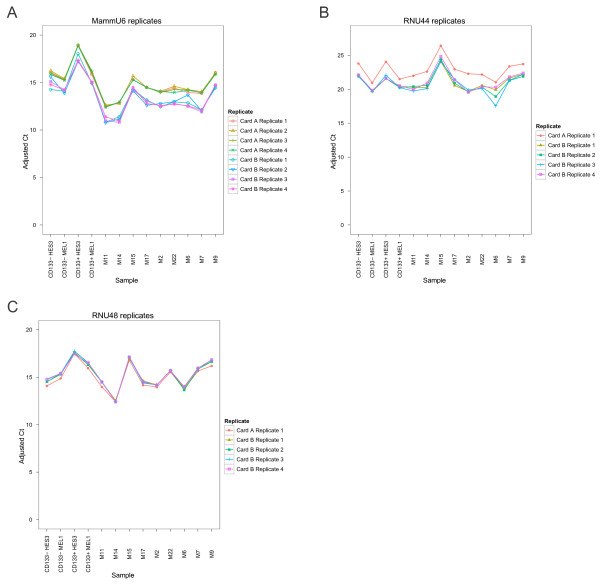
Quantification cycle (Cq) values for technical replicates of (A) MammU6 (B) RNU44 and (C) RNU48 across all samples for TaqMan low density array (TLDA) cards A and B.

miRNA profiling using the TLDA system consists of two cards, A and B. Card A contains three proposed EC references genes, MammU6, RNU44 and RNU48, and card B contains six, MammU6, RNU44, RNU48, RNU24, RNU43 and RNU6B. To evaluate whether there was inter-plate variation between the sn/snoRNA candidate EC reference genes on TLDA card A and card B, Cq values for technical replicates of three candidate EC reference genes, MammU6, RNU48 and RNU44, present on separate TLDA cards were plotted and one way analysis of variance (ANOVA) was applied to assess inter-plate variation between the technical replicates (Figure 1). Significant variation was observed between the technical replicates of MammU6 (*P* = 0.025) and RNU44 (*P* = 0.0030) on TLDA cards A and B (Figure 2). In contrast, Cq values for the technical replicates of RNU48 remained relatively stable (*P* = 0.97). The observed differences between EC reference gene replicate Cq values on TLDA cards A and B suggests that the normalisation of miRNAs is best performed to EC reference genes on the same TLDA card. Based on these results, we assessed the suitability of EC reference genes separately for TLDA cards A and B. As minimal variation was observed for technical replicates of candidate EC reference genes on the same TLDA card, an average of the technical replicates from each TLDA card was employed for further analysis. Because this approach limited the maximum number of proposed sn/snoRNA candidate EC reference genes for each TLDA card, particularly for TLDA card A, we expanded the panel of potential candidate EC reference genes under investigation to include miRNAs that fulfil the appropriate selection criteria outlined in methods.

### Expression of candidate EC reference genes in primary MB specimens, CD133+ NSCs and CD133- NPCs

Suitable candidate EC reference genes must display consistent expression between normal and treatment and/or disease groups. Two sample *t-*tests were employed to assess whether candidate EC reference genes were differentially expressed between primary MB specimens and CD133+ NSC/ CD133- NPC populations. For TLDA card A, 12 genes were identified as potentially suitable EC reference genes (Table 1). Of the proposed sn/snoRNA candidate EC reference genes, only RNU44 was uniformly expressed across primary MB specimens and CD133+ NSC/CD133- NPC populations (*P* = 0.71; fold change (FC) = 1.02) (Table 1). In contrast, MammU6 was over-expressed in primary MB specimens compared to NSC/NPCs (*P* = 0.049; FC = 3.66), and while the over-expression of RNU48 did not reach statistical significance (*P* = 0.35), the degree of over-expression observed in primary MB specimens relative to NSCs/NPCs (FC = 2.35) suggested RNU48 was not an ideal EC reference in this context. In addition to RNU44, a number of candidate miRNAs were uniformly expressed in NSCs and NPCs and primary MB specimens (*P* ≥ 0.05; FC ≤ ±1.5), highlighting their potential as EC reference genes in this study.

**Table 1:** Summary of candidate EC reference genes identified for the normalisation of miRNA expression data in MB

**Gene**	**Chromosomal location**	**p value**	**Fold change**	**Coefficient of variation**	**Candidate EC gene**
**Card A**					
RNU48	6p21.33	0.351	2.36	9.52	No
RNU44	1q25.1	0.709	1.02	6.50	Yes
MammU6	15q23	0.049	3.66	11.10	No
hsa-miR-133a	18q11.2/ 20q13.33	0.797	1.16	7.05	Yes
hsa-miR-339-5p	7p22.3	0.218	1.47	5.89	Yes
hsa-miR-214	1q24.3	0.853	1.36	8.45	Yes
hsa-miR-197	1p13.3	0.515	-1.41	5.65	Yes
hsa-miR-18a	13q31.3	0.687	1.09	9.64	Yes
hsa-miR-210	11p15.5	0.766	1.41	6.07	Yes
hsa-miR-328	16q22.1	0.564	1.46	7.40	Yes
hsa-miR-301a	17q22	0.384	1.32	7.23	Yes
hsa-miR-218	4p15.31	0.592	1.14	9.86	Yes
hsa-miR-149	2q37.3	0.647	-1.36	7.97	Yes
hsa-miR-20b	Xq26.2	0.397	-1.03	9.32	Yes
**Card B**					
RNU48	6p21.33	0.250	2.64	9.50	No
RNU44	1q25.1	0.968	1.49	6.61	Yes
MammU6	15q23	0.023	4.29	13.25	No
RNU24	9q34	0.423	1.19	4.77	Yes
RNU43	22q13	<0.01	498.00	21.50	No
RNU6B	10p13	<0.01	14.98	9.16	No
hsa-miR-425*	3q21.31	0.493	1.42	4.53	Yes
hsa-miR-877	6p21.33	0.350	1.39	3.33	Yes
hsa-miR-130b*	22q11.2	0.910	-1.13	6.39	Yes
hsa-miR-181a-2*	9q33.3	0.972	1.48	6.10	Yes

Uniformly and differentially expressed snoRNA candidate EC reference genes from both TLDA cards A and B are listed with chromosomal location.

Expression analysis identified six candidate EC reference genes of TLDA card B suitable for data normalisation (Table 1). Of the manufacturer-recommended candidate EC reference genes included on TLDA card B, no significant difference was observed for RNU44 (*P* = 0.97; FC = 1.49) and RNU24 (*P* = 0.42; FC = 1.19) between primary MB samples and NSCs/NPCs. Similar to findings obtained for TLDA card A, increased expression of RNU48 in primary MB specimens (FC = 2.64), although not statistically significant (*P* = 0.25), suggests it may not be the most appropriate EC reference for normalisation. Significantly increased expression of MammU6, RNU6B and RNU43 was identified in primary MB specimens relative to CD133+ NSCs/CD133- NPCs, particularly for RNU43 (~ 500 fold greater expression in primary MB). In addition to the two snoRNAs, RNU44 and RNU24, four miRNAs were also uniformly expressed in the two groups, namely miR-425* (*P* = 0.49; FC = 1.42), miR-877 (*P* = 0.35; FC = 1.39), miR-130b* (*P* = 0.91, FC = −1.13) and miR-181a-2* (*P* = 0.97; FC = 1.48).

**Figure 3:**
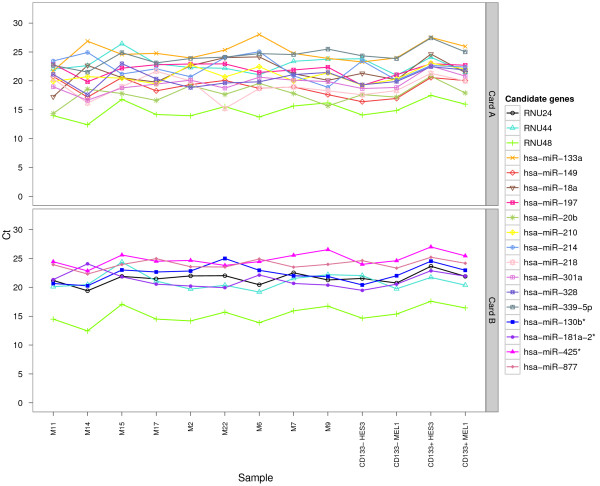
**Variation in expression of all candidate EC reference genes of (TLDA) card A and B identified as uniformly expressed between primary medulloblastoma (MB) specimens and CD133+ NSCs/ CD133- NPCs.** Quantification cycle (Cq) values for candidate EC reference genes were plotted for each sample. Cq values were also plotted for RNU48, as although differentially expressed, it did not reach statistical significance.

To compare the abundance of candidate EC reference genes selected for further analysis and to obtain an estimate of their uniformity of expression across CD133+ NSC/ CD133-NPCs and primary MB specimens, Cq values of each candidate EC reference gene were plotted and the coefficient of variation (CV) for each candidate EC reference gene calculated (Figure 3). The mean, CV and range of Cq values for all candidate EC reference genes are shown in Table 2. For TLDA card A, miR-20b had the highest expression with a mean Cq of 17.75, followed by miR-149 and miR-218 both with a mean Cq value of 18.88. Moderately abundant EC reference genes with mean Cq values between 20 and 25, included miR-18a, miR-133a, miR-197, miR-210, miR-214, miR-328 and RNU44. All candidates displayed low variability, with the least variation across all samples for miR-197 (CV = 5.65), and the largest for miR-218 (CV = 9.86). Candidate EC reference genes from TLDA card B were expressed at lower levels, with RNU44 the most highly expressed gene with a mean Cq value of 20.98. Closely following RNU44 were miR-181a-2* and RNU24, with mean Cq values of 21.22 and 21.53, respectively. miR-425* was the lowest expressed EC reference gene, with a mean Cq value of 24.87. Candidates again displayed low variability, with miR-877 exhibiting the smallest Cq variation (CV = 3.33), and RNU44 (CV = 6.60) the largest.

**Table 2:** Summary statistics of Cq values for candidate EC reference genes

**Gene**	**Mean**	**Minimum**	**Maximum**	**CV**
**Card A**				
RNU48	14.99	12.41	17.49	9.52
RNU44	22.85	20.96	26.43	6.50
hsa-miR-133a	24.97	21.74	28.02	7.05
hsa-miR-339-5p	24.28	21.52	27.43	5.89
hsa-miR-214	22.29	18.88	25.05	8.45
hsa-miR-197	21.94	19.20	22.97	5.65
hsa-miR-18a	21.54	17.25	24.64	9.64
hsa-miR-210	20.96	19.25	23.06	6.07
hsa-miR-328	20.50	17.61	22.98	7.40
hsa-miR-301a	19.56	16.64	22.58	7.23
hsa-miR-218	18.88	15.21	21.68	9.86
hsa-miR-149	18.88	16.39	20.77	7.97
hsa-miR-20b	17.75	14.36	20.81	9.32
**Card B**				
RNU48	15.30	12.45	17.58	9.50
RNU44	20.98	19.18	24.36	6.60
RNU24	21.53	19.40	23.64	4.77
hsa-miR-425*	24.87	22.83	26.99	4.53
hsa-miR-877	23.99	22.30	25.20	3.33
hsa-miR-130b*	22.39	20.26	24.99	6.39
hsa-miR-181a-2*	21.22	19.47	24.06	6.10

### Uniformity of candidate reference gene expression in primary MB specimens, CD133+ NSCs and CD133- NPCs

Candidate EC reference genes displaying similar expression levels in CD133+ NSC/ CD133- NPCs and primary MB specimens were further assessed for uniformity of expression across all samples using geNorm [36] and NormFinder [46] software. RNU48 was also included in this analysis, as although differential expression of this snoRNA, as measured by FC, was observed between primary MB specimens and CD133+ NSCs/ CD133- NPCs, this did not reach statistical significance. The chromosomal location of candidate EC reference genes was assessed to rule out any co-regulation of clustered miRNA genes transcribed as a single, polycistronic transcript (Table 1), as this would lead to an erroneous choice of an optimum EC gene reference pair using geNorm and NormFinder software. The ranking of the candidate EC reference genes determined by these programs is summarised in Table 3, and consistent results were obtained for both TLDA card A and card B. Normfinder and geNorm identified hsa-miR-301a and hsa-miR-339-5p as the two most uniformly expressed EC reference genes on TLDA card A, closely followed by hsa-miR-210 and RNU48. Normfinder identified the geometric mean of hsa-miR-339-5p and hsa-miR-301a as the most stable pair of EC reference genes, with a lower combined stability value of M = 0.116 when compared to the stability values of the EC reference genes alone (Table 3). This suggests that the combination of hsa-miR-339-5p and hsa-miR-301a should be used for data normalisation in preference to the single EC reference genes.

**Table 3:** Candidate EC reference genes ranked according to their expression stability as calculated by the geNorm and NormFinder algorithms

**Rank**	**Normfinder**		**geNorm**	
	**Gene**	**Stability**	**Gene**	**Stability (M)**
Card A				
1	hsa-miR-339-5p	0.16	hsa-miR-301a	1.589
2	hsa-miR-301a	0.16	hsa-miR-339-5p	1.603
3	RNU48	0.2	hsa-miR-210	1.634
4	hsa-miR-210	0.22	RNU48	1.680
5	hsa-miR-328	0.26	hsa-miR-197	1.726
6	hsa-miR-20b	0.28	hsa-miR-328	1.785
7	hsa-miR-197	0.3	hsa-miR-149	1.794
8	hsa-miR-133a	0.3	hsa-miR-20b	1.841
9	hsa-miR-149	0.31	hsa-miR-133a	1.954
10	hsa-miR-218	0.32	RNU44	2.074
11	hsa-miR-18a	0.390	hsa-miR-18a	2.169
12	RNU44	0.49	hsa-miR-218	2.188
13	hsa-miR-214	0.59	hsa-miR-214	2.505
Card B				
1	hsa-miR-425*	0.11	hsa-miR-425*	1.177
2	RNU24	0.11	RNU24	1.180
3	RNU48	0.16	RNU48	1.299
4	hsa-miR-877	0.23	hsa-miR-877	1.301
5	hsa-miR-130b	0.32	hsa-miR-130b	1.538
6	hsa-miR-181a-2*	0.34	RNU44	1.569
7	RNU44	0.39	hsa-miR-181a-2*	1.860

Of the candidate EC reference genes on TLDA card B, hsa-miR-425* and RNU24 were the two most uniformly expressed EC reference genes, followed by RNU48 and hsa-miR-877 (Table 3). The combination of hsa-miR-425* and RNU24 displayed a much lower stability value (M = 0.078) than either of the candidate EC reference genes alone, indicating that the geometric mean of these EC reference genes is the most suitable for miRNA expression normalisation of TLDA card B. Although both programs listed RNU48 in the top four most uniformly expressed candidate EC reference genes of TLDA card A and card B, it was not considered ideal due to its differential expression between primary MB specimens and CD133+ NSCs/CD133- NPCs.

### Impact of EC reference genes on the relative quantification of individual miRNAs

**Figure 4:**
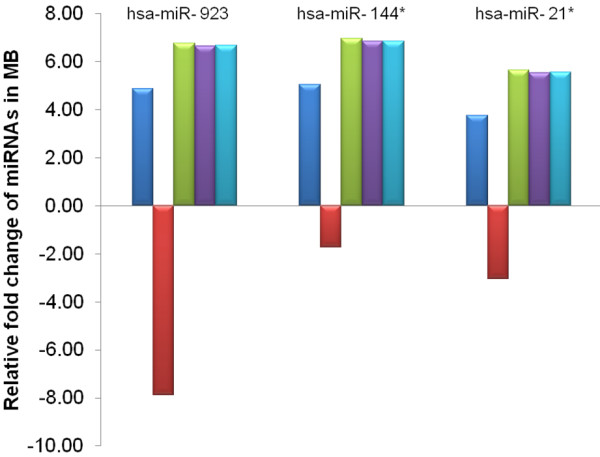
**Quantitative differences in miRNA expression in medulloblastoma (MB) normalising to different EC reference genes.** The mean fold changes of hsa-miR-923, hsa-miR-144* and hsa-miR-21* expression in nine primary MB specimens in comparison to the mean expression of these miRNAs in CD133+ NSCs and CD133- NPCs. Relative expression was determined using the 2^-ΔCq^ method and log_2_ transformed. Normalisation was carried out either to hsa-miR-425* (dark blue), RNU43 (red), hsa-miR-877 (green), RNU24 (purple) or geometric mean of hsa-miR-425* and RNU24 (light blue). The fold changes of all three miRNAs normalised against RNU43 was different from the fold changes obtained following the normalisation against hsa-miR-425*, hsa-miR-877, RNU24 and the geometric mean of hsa-miR-425* and RNU24.

To demonstrate the impact of selected EC reference genes on the results, we measured the expression of miRNAs relative to several candidate EC reference genes identified in this investigation. Specific candidate snoRNA EC reference genes on TLDA card B were highly differentially expressed in primary MB specimens relative to CD133+ NSCs and CD133- NPCs, and therefore, as proof of concept, the normalisation of individual miRNAs focused upon TLDA card B miRNAs and candidate EC reference genes. In primary MB specimens, the over-expression of three TLDA card B miRNAs, hsa-miR-144*, hsa-miR-21* and hsa-miR-923, was previously described [47]. For this analysis, normalisation of these three miRNAs was performed using top ranked candidate EC reference genes identified by geNorm and Normfinder, including hsa-miR-425*, RNU24, hsa-miR-877, and the EC reference pair, RNU24/ hsa-miR-425*. In addition, target miRNA expression was also established relative to the significantly over-expressed (~500 fold) candidate EC reference gene, RNU43. Normalisation using uniformly expressed candidate EC reference genes did not influence the directionality of differential expression of miRNAs, with consistent over-expression of each target miRNA observed in primary MB specimens relative to CD133 + NSCs/CD133- NPCs (Figure 4). In contrast, down-regulation of all three miRNAs was observed when RNU43 was utilised for data normalisation, due to the significant over-expression of RNU43 in primary MB specimens relative to target miRNA expression. Taken together, these results emphasise that establishing target miRNA expression levels for a particular tissue and/or disease state is strongly influenced by the candidate EC reference gene chosen for data normalisation.

## Discussion

The importance of validating suitable candidate EC reference genes in a cell and/or tissue-specific context is well documented, and a single universal reference gene for all tissue types is unlikely to exist [33-37]. Indeed, in the context of cancer, a disease in which the cell of origin is unknown or inadequately characterised in many cases, the selection of appropriate EC reference genes is particularly difficult. Although the cells of origin of the at least four molecular sub-types of human MB have not been conclusively identified, the available murine data suggest that SHH-dependent MB may have multiple cells of origin including cerebellar granule precursor cells, NSCs [48,49], and cochlear nuclei of the lower rhombic lip [50]. Other mouse models suggest that Group 3 MB arise from CD133+ NSCs [11], and WNT-driven tumours originate from progenitor cells of the dorsal brainstem [51]. The cell of origin of Group 4 tumors has not yet been determined. On this basis, the normalisation of gene expression levels in primary MB specimens to levels in whole foetal or adult cerebellum, which represent heterogeneous tissues at different developmental stages, although generally accepted, is likely to be sub-optimal. Similarly, the normalisation of gene expression levels in primary human MB to those in ESC-derived NSCs as described here, may be more appropriate for the analysis of specific MB subtypes (e.g., SHH or Group 3 MB), or stem cell regulatory pathways in MB pathogenesis. The assessment of miRNA expression levels in a larger cohort of primary MB specimens relative to NSCs, and an improved understanding of MB cells of origin will be necessary to address these issues. In the meantime, the data reported here represent an important complementary resource for future MB miRNA profiling studies.

Normalisation based on predefined invariant EC genes such as sn/snoRNAs is a commonly utilised approach in miRNA RT-qPCR profiling analysis [52]. However, in the context of this study, the majority of pre-defined small nuclear and small nucleolar RNAs were not suitable as EC reference genes for miRNA data normalisation. Small non-coding RNAs other than miRNAs do not mirror the physiochemical properties of miRNA molecules, suggesting that normalisation of miRNA expression data should be performed with reference genes belonging to the same RNA class [33,36]. The selection of invariant miRNAs identified by algorithms specifically tailored to reference gene ranking by stepwise elimination of the least stable gene such as geNorm [36], or through statistical linear mixed-effects modelling such as Normfinder [46] have been previously identified as superior to sn/snoRNA-based normalisation [33,53]. Indeed, we identified various miRNAs that were more uniformly expressed across all samples and therefore represent more suitable EC reference genes than the sn/snoRNAs proposed by the manufacturer, a finding that was consistent with a number of previous studies [38,54-57]. Thus, our data provide further evidence to suggest that miRNAs, in comparison to other classes of small non-coding RNAs, may be more suitable EC reference genes for the normalisation of miRNA expression data, providing their expression meets the general consensus of moderate abundance and consistency across all samples.

The overall uniformity of expression is a major determinant for an ideal EC reference gene [33]. Our initial statistical analyses identified a number of candidate EC reference genes uniformly expressed across experimental groups, with both NormFinder and geNorm identifying hsa-miR-339-5p and hsa-miR-301a as the most stable EC reference genes for TLDA card A, and hsa-miR-425* and RNU24 as the most stable for TLDA card B. The practical consequences of miRNA normalisation were then evaluated using TLDA card B candidate EC reference genes and miRNAs as a case study. As evident from our results, inappropriate use of EC reference genes can significantly impact upon target miRNA quantitation. With the use of suitable EC reference genes, including hsa-miR-425*, RNU24, hsa-miR-877 and EC reference pair, RNU24/hsa-miR-425*, over-expression of all three miRNAs (hsa-miR-21*, hsa-miR-144* and hsa-miR-923) was identified, as previously reported [47]. However, when an inappropriate EC reference gene, RNU43, was used for expression data normalisation, the down-regulation of all three miRNAs was observed. Several previously published studies in a range of other tissue types have reported similar misleading results when inappropriate EC reference genes were used [33,38,54,55,58], highlighting the importance of selecting appropriate and validated EC reference genes for miRNA expression data normalisation. Although several previous MB miRNA profiling studies utilised RNU6B and RNU66 as an EC reference gene pair for normalisation, the validation of these reference genes was not reported [39,41,45]. It is important to note that these previous studies normalised miRNA expression to EC reference levels in human normal adult and foetal cerebellum, rather than CD133+ NSCs/CD133- NPCs profiled in this investigation. The findings obtained in this study indicated that RNU6B was significantly differentially expressed in primary MB specimens relative to CD133+ NSCs/CD133- NPCs, and therefore was not a suitable EC reference gene for normalisation. Unfortunately, the suitability of RNU66 could not be assessed in this investigation, as the RNU66 miRNA assay was not included in the V2.0 TLDA cards. An additional MB miRNA profiling study utilised RNU48 as a single reference gene for normalisation of miRNA expression data [44]. Differential expression of RNU48 was observed in this study, however this did not reach statistical significance and again, a direct comparison was not possible due to the different normal control tissues being profiled. Combined, these findings reiterate the importance of validating suitable candidate EC reference genes in the relevant cell and/or tissue under investigation.

The performance of miRNA profiling by the high-throughput TLDA miRNA expression profiling system has been evaluated in several studies, with high reproducibility observed for technical replicates of miRNA assays located on the same TLDA card [53,59-61]. While we also observed minimal intra-card variability for all candidate EC reference genes, significant inter-card variability was apparent between replicates of MammU6 and RNU44 on TLDA cards A and B. Similar findings were obtained in a previous study, where differential expression of MammU6 and RNU44, but not RNU48, was observed between the same samples profiled on TLDA cards A and B [61]. For the TLDA system, two separate Megaplex primer pools are required for reverse transcription (RT) of RNA and pre-amplification of cDNA prior to application to cards A and B. We propose that the inter-card variability for replicates of MammU6 and RNU44 was perhaps a direct consequence of differential RT and pre-amplification efficiencies of these sn/snoRNAs associated with the separate reactions. Previously, the variable expression of a subset of miRNAs was largely attributed to the altered efficiencies of the Megaplex RT and pre-amplification reactions [53]. Whilst a systematic evaluation of the inter-card reproducibility of the miRNA TLDA platform has not been reported, the findings obtained in this study have important implications for the normalisation of miRNA expression data obtained using this system. Future studies should carefully assess the potential for inter-card/plate variability of the associated platform prior to the assessment of candidate EC reference genes for data normalisation.

## Conclusion

A panel of 18 potential EC reference genes that were not significantly differentially expressed between CD133+ NSCs/ CD133- NPCs and primary human MB specimens was identified. Based on our findings, EC reference gene pairs hsa-miR-301a and hsa-miR-339-5p and hsa-miR-425* and RNU24 are recommended for the normalisation of miRNAs on TLDA card A and card B, respectively, in primary MB specimens relative to ESC-derived NSCs/ NPCs. The top ranked EC reference genes described here should be validated in a larger cohort of specimens to verify their utility as controls for the normalisation of RT-qPCR data generated in MB miRNA expression studies. More broadly, inter-card variability observed between replicates of certain candidate EC reference genes has major implications for the accurate normalisation of miRNA expression data obtained using the miRNA TLDA platform.

## Methods

### Patient samples

Nine MB samples were collected from children treated at Princess Margaret Hospital (PMH) in Perth, Western Australia. Tumour tissue was embedded in optimal cutting temperature compound (OCT) and snap-frozen. The age of patients, gender distribution and molecular sub-type of each primary MB specimen have been previously described (Table 4) [47]. Written approval to undertake this study was obtained from the PMH human ethics committee. Written consent to use tumour material for research purposes was obtained from the parents of patients according to PMH ethics committee guidelines. All tumour material was de-identified to ensure patient anonymity.

**Table 4:** Clinical data and molecular sub-type of primary MB specimens utilised in this investigation

**Sample**	**Gender**	**Age**	**M Status**	**Sub-type**
M2	Female	3	Unknown	B
M6	Male	7	M+	C
M7	Male	1.5	M0	B
M9	Male	2	Unknown	N/A
M11	Male	5	M+	DE
M14	Female	3	M0	C
M15	Male	3	M+	DE
M17	Male	4	M+	DE
M22	Male	1	Unknown	N/A

Metastatic status was defined as M0 (no metastasis) or M + (distant metastases). Primary specimens defined as “N/A” were not available for sub-typing analysis [47]. Sub-type designations were based on the data of Kool *et al*., [3].

### Neurosphere maintenance and flow cytometry

Human NSCs propagated as neurospheres were derived from human embryonic stem cell (ESC) lines hES3 (WiCell Research Institute, Madison, WI, USA) and MEL1 (StemCore, Melbourne, Australia) using protocols described previously [62,63]. Dissociation of neurospheres and isolation of CD133+ NSCs by flow cytometry was performed as described previously [64]. Enrichment of CD133- NPCs was 100% for both hES3 and MEL1 ESC lines, with the enrichment of CD133+ NSCs being 81.1% and 97% for the hES3 and MEL1 ESC lines, respectively.

### Small RNA isolation and enrichment

RNA enriched for small RNAs was isolated from primary specimens, cell lines and NSC/NPCs using the miRNeasy mini kit (Qiagen, Melbourne Australia), as described previously [47]. RNA quantity and purity was estimated by the ratio of absorbance at 260 nm to that at 280 nm (OD_260_:OD_280_), with ratios of between 1.8 and 2.0 being considered optimal.

### miRNA expression profiling

miRNA profiling was performed using quantitative real-time RT-PCR (RT-qPCR) utilising pre-printed TaqMan low density array (TLDA) microfluidic cards (Human miR v2.0, Applied Biosystems) as described [47]. Each TLDA card set contained MGB-labelled probes specific to 662 mature miRNAs plus six proposed small nuclear (sn)/ small nucleolar (sno) RNA candidate EC reference genes (MammU6, RNU44, RNU48, RNU24, RNU43, RNU6B). Assays for three of these candidate EC reference genes (MammU6, RNU44 and RNU48) were included on TLDA cards A and B, and the other three (RNU24, RNU43 and RNU6B) were only present on TLDA card B. Details of these candidate EC reference genes and the number of technical replicates found on TLDA cards A and B are provided in Table 5. Pre-processing of raw TLDA data files consisted of threshold and baseline corrections for each sample, with each amplification plot assessed to confirm that the quantification cycle (Cq) value corresponded with the midpoint of logarithmic amplification (SDS 2.3, Life Technologies, Melbourne). Cq values greater than 32 were imputed to 32 according to the manufacturer’s technical recommendation.

**Table 5:** Small nuclear and small nucleolar RNA candidate EC reference genes included on TLDA cards

**Gene**	**Length (nt)**	**RNA species**	**Entrez Gene ID**	**Replicates on Card A**	**Replicates on Card B**	**Total number of replicates**
RNU48	63	snoRNA	NR_002745	1	4	5
RNU44	61	snoRNA	NR_002750	1	4	5
RNU43	62	snoRNA	NR_002439	0	4	4
RNU24	75	snoRNA	NR_002447	0	4	4
RNU6B	45	snoRNA	NR_002752	0	4	4
MammU6	106	snRNA	NR_004394	4	4	8

Accession numbers for each of the candidate EC reference genes and number of replicates per card are also listed.

### Statistical analysis

Statistics used for the filtering of candidate EC reference genes from TLDA cards were calculated in R statistical environment version 2.13.0 [65] and candidates were filtered according to three criteria: (1) candidate EC reference genes must be moderately to highly expressed across all samples, defined as a mean Cq of ≤ 25; (2) candidate EC reference genes must be consistently expressed between the combined CD133+ NSCs and CD133- NPCs, and primary MB specimens, with significantly differentially expressed miRNAs identified using a two sample *t-*test (*P* < 0.05) and a absolute differential expression fold change (FC) value of 1.5 ( |FC| ≥ 1.5); (3) candidate EC reference genes must be uniformly expressed across all samples, with a coefficient of variation (CV) < 10. The coefficient of variation (CV) is the ratio of the standard deviation to the mean expressed as a percentage and is a measure of expression variability of candidate EC reference genes across all samples. Candidate EC reference genes that met these criteria (see Table 2) were deemed suitable for subsequent analysis using the geNorm [36] and NormFinder [46] software. The geNorm algorithm calculates the gene expression stability measure (M) for a candidate EC reference gene based upon the average pairwise variation (V) for that gene against all other tested candidate EC reference genes [36]. NormFinder, a Microsoft Excel add-in, is based upon an analysis of variance (ANOVA) model which estimates an intra- and inter-group variation to provide a stability value for each candidate EC reference gene [46]. Normfinder provides the single most stable reference gene, in addition to an EC reference gene pair that has a stability value less than that of the single EC reference gene. Prior to NormFinder analyses, Cq values were converted to relative expression values using the 2^-Cq^ method [66]. In both programs, lower values indicate increased stability of EC reference genes, and therefore allow for the ranking of genes on this basis. Cq values of the target TLDA card B miRNAs hsa-miR-923, hsa-miR-144* and hsa-miR-21* were further normalised to each selected EC reference gene and relative expression was determined using the 2^-ΔCq^ method, where ΔCq = (Cq_miR_ – Cq_endogenous control gene_). To identify differential expression of individual miRNAs, the normalised means [log_2_(2^-ΔCq^)] of expression levels for each miRNA in primary MB specimens were compared to the normalised means (log_2_(2^-ΔCq^) of expression for that miRNA in CD133+ NSCs and CD133-NPCs. For CD133+ NSCs and CD133- NPCs, normalised means (log_2_(2^-ΔCq^) of expression levels were obtained by averaging the expression of individual miRNAs from both hES3 and Mel1, therefore representing a pool of two ESC cell lines.

### Availability of supporting data

The data sets supporting the results of this article are available from the Telethon Institute for Child Health Research (TICHR) repository: http://bioinformatics.childhealthresearch.org.au/datasets/.

## Competing interests

The authors have declared that no competing interests exist.

## Authors’ contributions

Conceived and designed the experiments: LAG DA KMG PBD, Performed the experiments: LAG. Analysed the data: LAG DA, Contributed reagents/materials/analysis tools: PBD, KWC, Wrote the paper: LAG DA KWC KMG PBD. All authors read and approved the final manuscript.
